# Early Prediction of Functional Outcomes After Acute Ischemic Stroke Using Unstructured Clinical Text: Retrospective Cohort Study

**DOI:** 10.2196/29806

**Published:** 2022-02-17

**Authors:** Sheng-Feng Sung, Cheng-Yang Hsieh, Ya-Han Hu

**Affiliations:** 1 Division of Neurology Department of Internal Medicine Ditmanson Medical Foundation Chia-Yi Christian Hospital Chiayi City Taiwan; 2 Department of Nursing Min-Hwei Junior College of Health Care Management Tainan Taiwan; 3 Department of Neurology Tainan Sin Lau Hospital Tainan Taiwan; 4 Department of Information Management National Central University Taoyuan City Taiwan

**Keywords:** acute ischemic stroke, bag-of-words, extreme gradient boosting, machine learning, MetaMap, natural language processing, outcome prediction, text classification, unstructured clinical text

## Abstract

**Background:**

Several prognostic scores have been proposed to predict functional outcomes after an acute ischemic stroke (AIS). Most of these scores are based on structured information and have been used to develop prediction models via the logistic regression method. With the increased use of electronic health records and the progress in computational power, data-driven predictive modeling by using machine learning techniques is gaining popularity in clinical decision-making.

**Objective:**

We aimed to investigate whether machine learning models created by using unstructured text could improve the prediction of functional outcomes at an early stage after AIS.

**Methods:**

We identified all consecutive patients who were hospitalized for the first time for AIS from October 2007 to December 2019 by using a hospital stroke registry. The study population was randomly split into a training (n=2885) and test set (n=962). Free text in histories of present illness and computed tomography reports was transformed into input variables via natural language processing. Models were trained by using the extreme gradient boosting technique to predict a poor functional outcome at 90 days poststroke. Model performance on the test set was evaluated by using the area under the receiver operating characteristic curve (AUC).

**Results:**

The AUCs of text-only models ranged from 0.768 to 0.807 and were comparable to that of the model using National Institutes of Health Stroke Scale (NIHSS) scores (0.811). Models using both patient age and text achieved AUCs of 0.823 and 0.825, which were similar to those of the model containing age and NIHSS scores (0.841); the model containing preadmission comorbidities, level of consciousness, age, and neurological deficit (PLAN) scores (0.837); and the model containing Acute Stroke Registry and Analysis of Lausanne (ASTRAL) scores (0.840). Adding variables from clinical text improved the predictive performance of the model containing age and NIHSS scores, the model containing PLAN scores, and the model containing ASTRAL scores (the AUC increased from 0.841 to 0.861, from 0.837 to 0.856, and from 0.840 to 0.860, respectively).

**Conclusions:**

Unstructured clinical text can be used to improve the performance of existing models for predicting poststroke functional outcomes. However, considering the different terminologies that are used across health systems, each individual health system may consider using the proposed methods to develop and validate its own models.

## Introduction

Stroke is a common and serious neurologic disorder. Approximately 1 out of every 4 adults aged ≥25 years will experience a stroke in their lifetime [1]. Despite recent and emerging advances in the acute treatment of strokes, more than half of patients with stroke still experience an unfavorable outcome, which can result in permanent functional dependency or even death [2]. In clinical practice, having a handy and readily available prognostic tool is desirable for clinical decision-making and resource allocation. Prognostic understanding is of direct clinical relevance and is essential for informing goals-of-care discussions. It also facilitates discharge planning, communication, and postdischarge support.

Several prognostic scores have been developed to predict functional outcomes following an acute stroke. Most of these scores use similar input variables for their predictions. As functional outcomes are largely determined by age and stroke severity [3], these two variables are almost always included in existing prognostic scores [4]. Other commonly used input variables may include comorbidities, neurologic status, and biochemical parameters. For example, the preadmission comorbidities, level of consciousness, age, and neurological deficit (PLAN) score [5] includes comorbidities (preadmission dependence, cancer, congestive heart failure, and atrial fibrillation) and neurologic focal deficits (weakness of the leg or arm, aphasia, or neglect) as additional predictors. The Acute Stroke Registry and Analysis of Lausanne (ASTRAL) score [6] comprises age, stroke severity, stroke onset to admission time, the range of visual fields, acute glucose level, and the level of consciousness. However, the feasibility of these scores in daily clinical practice and their relevance to a specific clinical setting need to be well thought out prior to implementation [4]. Furthermore, using structured information alone, as well as the almost universal use of logistic regression models in the development of traditional prognostic scores [4,7], which require the assumption that linear and additive relationships are being fulfilled among predictors, significantly limits the applicability of these prognostic scores to an individual hospital or health system [8].

The ubiquitous use of electronic health records (EHRs) and the increase in computational power provide an opportunity to incorporate various types of structured data for the data-driven prediction of important clinical outcomes [9]. Machine learning algorithms have been used to develop prognostic models to predict various poststroke outcomes [10-16]. In previous studies that aimed to predict functional outcomes after an acute ischemic stroke (AIS), data-driven machine learning models generally performed equally as well as the PLAN and ASTRAL scores [10-12]. Matsumoto et al [10] developed and validated data-driven models via linear regression or decision tree ensembles and also validated traditional prognostic scores. Although no direct statistical comparisons of predictive performance were made between models, they concluded that data-driven models may be alternative tools for predicting poststroke outcomes. Monteiro et al [11] found that machine learning models, including decision tree ensembles and support vector machines, achieved only a marginally higher predictive performance than that of traditional prognostic scores. Finally, Heo et al [12] found that machine learning models developed via random forest and logistic regression had a similar predictive performance to that of the ASTRAL score, while the deep neural network model outperformed this traditional prognostic score.

In addition to structured data, EHRs store a multitude of unstructured textual data, such as narrative clinical notes, radiology reports, and pathology reports. To our knowledge, this kind of information has not been explored in the development of stroke prognostic models [10-16]. However, natural language processing (NLP) has been used to extract valuable information stored in textual data within other medical applications. By harnessing the information from textual data, it is possible to improve the prognostication of patients with critical illness [8] and the detection of severe infection during emergency department triage [17]. Motivated by these ideas, we aimed to investigate whether machine learning models using unstructured clinical text can improve the prediction of functional outcomes at an early stage after AIS.

## Methods

### Study Settings

Data that support the study findings are available from the corresponding author on reasonable request. This retrospective study was conducted in a 1000-bed teaching hospital that had a catchment area with around 500,000 inhabitants. The stroke center of this hospital has been prospectively registering all patients who are hospitalized for a stroke and collecting data that conform to the design of the nationwide Taiwan Stroke Registry [18] since 2007. Data on patient demographics, personal and medical histories, stroke severity as assessed by using the National Institutes of Health Stroke Scale (NIHSS), the treatments that patients received, hospital courses, and final diagnoses were collected. Follow-up data, such as functional outcomes as assessed by the modified Rankin Scale (mRS), were collected only from patients who gave written informed consent for the follow-up evaluation.

### Ethics Approval

The study protocol was approved by the Ditmanson Medical Foundation Chia-Yi Christian Hospital Institutional Review Board (approval number: CYCH-IRB 2020090). Study data were maintained with conﬁdentiality to ensure the privacy of all participants.

### Study Population

We identified all consecutive adult patients who were admitted to the study hospital for the first time for AIS from October 2007 to December 2019 by using the institutional stroke registry. Patients who experienced an in-hospital stroke or those who were missing admission NIHSS scores from their clinical data were excluded. Those who did not provide consent for the follow-up or were lost to follow-up at 90 days were also excluded. For each patient, we retrieved the history of present illness (HPI) upon admission and the initial computed tomography (CT) report from the EHR database. Patients whose EHRs were unavailable were excluded.

To train and evaluate the machine learning models, we split the study population randomly into a training set that consisted of 75% (2885/3847) of the patients and a holdout test set that consisted of the remaining 25% (962/3847) of the patients, who were withheld from all models during the training process.

### Outcome Variable

The outcome of interest was a poor functional outcome as assessed by using the mRS score 90 days after a stroke. The mRS score was dichotomized into a good outcome (mRS score of 0-2) versus a poor outcome (mRS score of 3-6).

### Text Vectorization and Feature Selection

**Figure 1:**
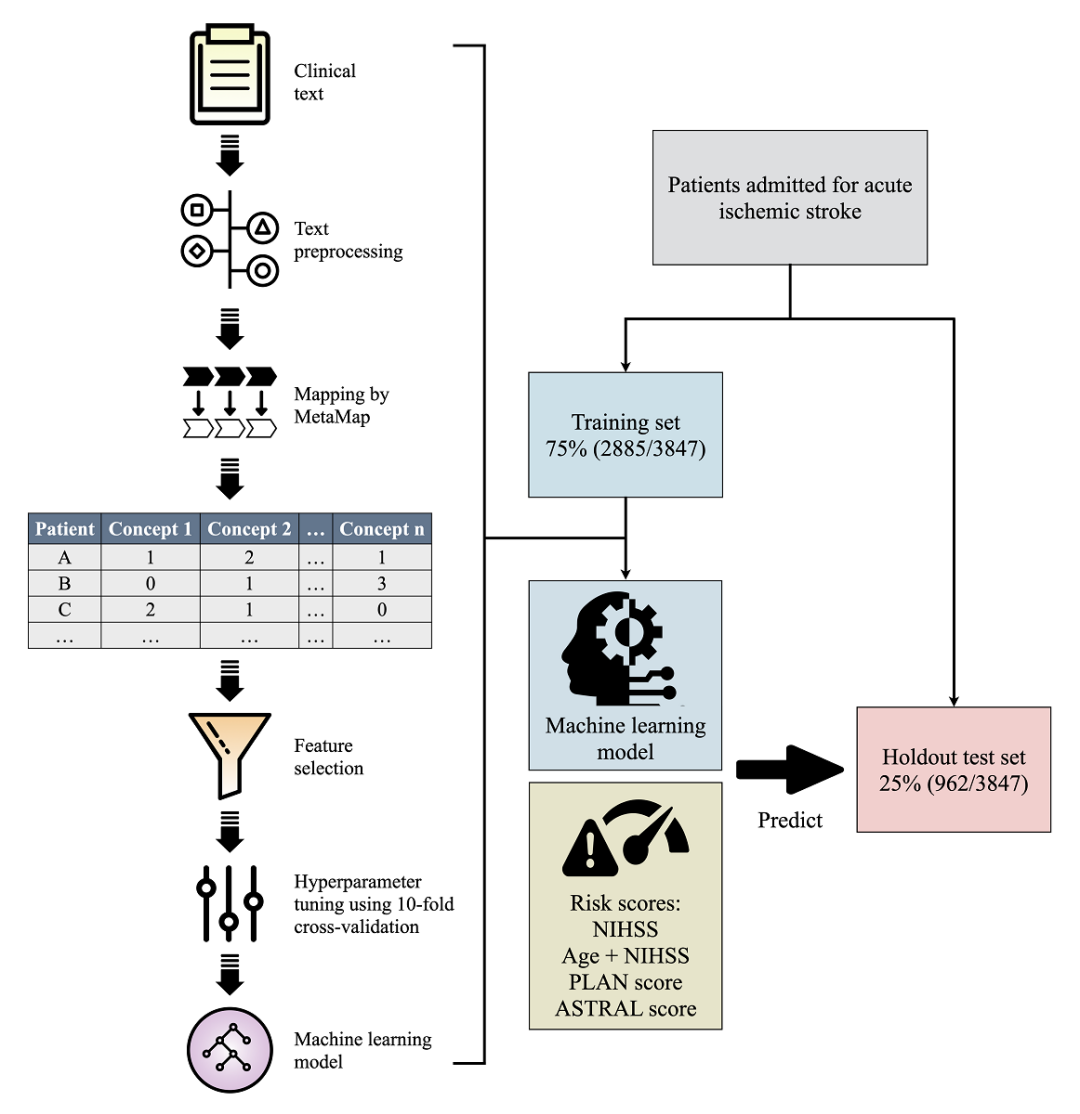
Model development and validation. ASTRAL: Acute Stroke Registry and Analysis of Lausanne; NIHSS: National Institutes of Health Stroke Scale; PLAN: preadmission comorbidities, level of consciousness, age, and neurological deficit.

The model development and validation process is illustrated in Figure 1. The free text extracted from the HPIs and CT reports was processed separately by using the following NLP techniques: (1) misspelled words were corrected by using the Jazzy spellchecker [19]; (2) abbreviations and acronyms were expanded to their full forms by looking up a list of common clinical abbreviations and acronyms, which is maintained by the stroke center of the study hospital (Multimedia Appendix 1); and (3) non-ASCII (American Standard Code for Information Interchange) characters and nonword symbols were removed.

After text preprocessing, we used MetaMap to identify medical concepts from clinical text. MetaMap is an NLP tool that was developed by the National Library of Medicine [20]. Through the process of tokenization, sentence boundary determination, part-of-speech tagging, and parsing, input text was decomposed and transformed to variants of words or phrases, which were mapped to medical concepts in the Unified Medical Language System Metathesaurus. MetaMap was configured with the option of using the NegEx algorithm to identify negated concepts. We appended the suffix *_Neg* to concepts that were identified as negated. Next, the clinical text was vectorized for the text classification task by using the bag-of-words approach [21] or, more specifically, the so-called *bag-of-concepts approach* [22]. We built a document-term matrix in which each column represented each unique feature (concept) from the text corpus, the rows represented each document (the HPI or CT report for each patient), and the cells represented the counts of each concept within each document.

To reduce the number of redundant and less informative features and to improve training efficiency [21], we performed feature selection by filtering out concepts that appeared in less than 5% (145/2885) of all documents in the training set and then used 1 of the following 2 feature selection methods. The first method involved performing a penalized logistic regression with 10-fold cross-validation to identify the most predictive concepts [8,23]. The second involved using an extra tree classifier to determine important concepts based on the Gini index [24]. A large number of predictor variables (concepts) were still retained in the feature vector after these steps. To develop more parsimonious models, we built another document-term matrix by selecting the top 20 concepts that appeared in the documents of patients with poor or good functional outcomes based on chi-square statistics [25]. The same feature selection procedures were applied to the parsimonious models.

### Development of Machine Learning Models

Extreme gradient boosting (XGBoost) is an extension of gradient boosting algorithms [26]. It is an ensemble of classification and regression trees that can capture nonlinear interactions among input variables. The XGBoost algorithm trains a series of trees in which each subsequent tree attempts to correct the errors of the prior trees. XGBoost has gained popularity for predictive modeling in the medical field because of its high performance and scalability [24,27,28]. The XGBoost algorithm was implemented in Python 3.7 with xgboost Python package version 0.90.

**Figure 2:**
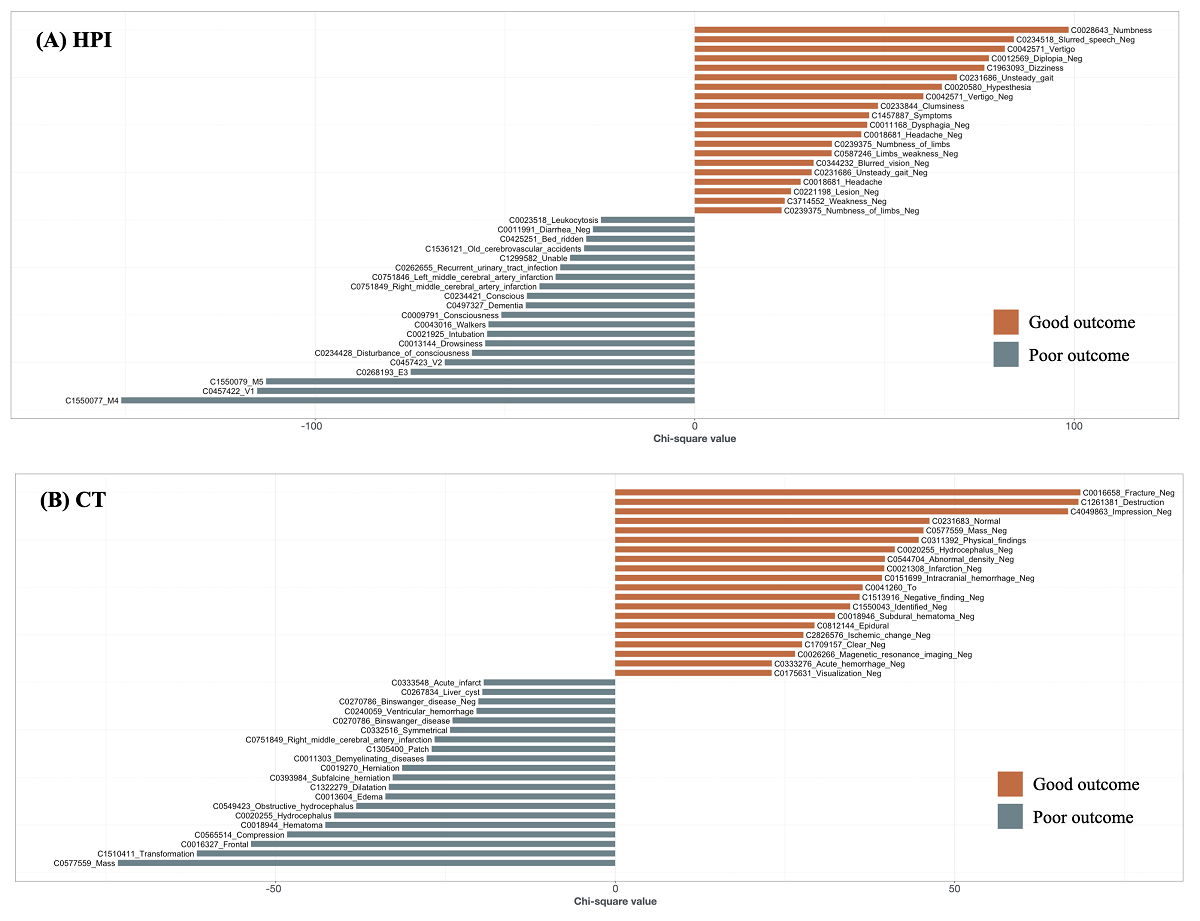
Keyness plots showing the top 20 concepts that frequently appear in the (A) HPIs and (B) CT reports of patients with good or poor functional outcomes. The prefix before the concept is the concept unique identifier. A negated concept is suffixed with “_Neg.” CT: computed tomography; HPI: history of present illness.

We built 6 text-based models for predicting poor functional outcomes by using the XGBoost algorithm. Full model 1 was trained by using the features derived from the HPIs. Full model 2 was trained by using the features derived from both the HPIs and CT reports. In addition to the features used in full model 2, full model 3 included patient age as an input variable. Simple model 1 was trained by using only the selected concepts from the HPIs (Figure 2), and simple model 2 was trained by using the selected concepts from both the HPIs and CT reports (Figure 2). Similarly, simple model 3 included patient age.

Hyperparameter optimization for each model was performed by repeatedly performing 10-fold cross-validation 10 times on the training set. We followed the steps proposed in a previous study [24] and conducted a grid search to find optimal hyperparameters. Model error was minimized in terms of the area under the receiver operating characteristic curve (AUC). Once the optimal hyperparameters were determined, the final models were fitted with the full training set.

With the introduction of machine learning techniques into health care settings, machine learning–based prediction models are being used to assist health care providers in decision-making for diagnosis, risk stratification, and clinical care. For decisions of such importance, clinicians prefer to know the reasons behind predictions rather than use a black-box model for prediction. The interpretability of model predictions is therefore considered a high priority for the implementation and use of prediction models [29]. To this end, after building the text-based models, we used Shapley additive explanations (SHAPs) [30], which are based on classic Shapley values from game theory, to explain the output of the XGBoost classifiers.

### Traditional Prognostic Models

A total of 4 traditional prognostic models based on the clinical data that were available at the time of admission were chosen for experimentation. The model using NIHSS scores served as the first baseline model. The second baseline model consisted of age and NIHSS scores [3]. The third baseline model consisted of the PLAN scores [5]. The fourth baseline model consisted of the ASTRAL scores [6].

### Statistical Analysis

Categorical variables were expressed as counts and percentages, while continuous variables were expressed as means with SDs or medians with IQRs. Differences between groups were tested by using chi-square tests for categorical variables and 2-tailed *t* tests or Mann-Whitney *U* tests for continuous variables, as appropriate.

Model performance was evaluated on the test set. For each patient in the test set, the probability of a poor functional outcome was generated by using the six text-based machine learning models. To assess the predictive performance of each of the baseline models and text-based models, a logistic regression was used to predict a poor functional outcome. Furthermore, to assess the added usefulness of information from the clinical text, the output (the probability of a poor functional outcome) of simple model 2, which was based on unstructured clinical text from the HPIs and CT reports, was treated as an additional continuous variable and added to the baseline models. Discriminatory ability was evaluated by calculating AUCs. The differences in AUCs among the models were compared by using the DeLong method [31]. In addition, improvements in predictive performance resulting from the addition of information from clinical text to each baseline model was evaluated by calculating the continuous net reclassification improvement and integrated discrimination improvement indices, as described by Pencina et al [32,33].

All statistical analyses were performed by using Stata 15.1 (StataCorp LLC) and R version 3.6.2 (R Foundation for Statistical Computing). Further, 2-tailed *P* values were considered statistically significant at <.05.

## Results

A total of 6176 patients were admitted for AIS. After excluding those with an in-hospital stroke (n=186), those who were missing clinical data (n=216), those who did not consent to the follow-up or were lost to follow-up (n=1048), and those with unavailable EHRs (n=295), the remaining 3847 patients comprised the study population. Of these, 1674 (43.5%) had a poor functional outcome after 90 poststroke days. Patients with a poor functional outcome were older, were more likely to be female, had more comorbidities (excluding hyperlipidemia), and were more likely to be dependent before the stroke. Stroke severity, PLAN scores, and ASTRAL scores were significantly higher among those with a poor functional outcome (Table 1).

**Table 1:** Baseline characteristics of the study population.

Characteristics	All (N=3847)	Functional outcome			*P* value
		Good (n=2173)	Poor (n=1674)		
Age (years), mean (SD)	69.5 (12.3)	66.1 (11.9)	74.0 (11.4)	<.001	
Female, n (%)	1583 (41.1)	771 (35.5)	812 (48.5)	<.001	
Hypertension, n (%)	3098 (80.5)	1694 (78)	1404 (83.9)	<.001	
Diabetes mellitus, n (%)	1602 (41.6)	846 (38.9)	756 (45.2)	<.001	
Hyperlipidemia, n (%)	2195 (57.1)	1323 (60.9)	872 (52.1)	<.001	
Atrial fibrillation, n (%)	684 (17.8)	246 (11.3)	438 (26.2)	<.001	
Congestive heart failure, n (%)	196 (5.1)	68 (3.1)	128 (7.6)	<.001	
Cancer, n (%)	249 (6.5)	106 (4.9)	143 (8.5)	<.001	
Preadmission dependence (mRS^a^ score of >2), n (%)	419 (10.9)	29 (1.3)	390 (23.3)	<.001	
Onset-to-admission delay (>3 hours), n (%)	2763 (71.8)	1574 (72.4)	1189 (71)	.34	
NIHSS^b^ score, median (IQR)	5 (3-10)	4 (2-6)	10 (5-19)	<.001	
Glucose (mg/dl), mean (SD)	163 (83)	161 (82)	166 (84)	.06	
PLAN^c^ score, median (IQR)	8 (6-12)	7 (6-8)	12 (9-17)	<.001	
ASTRAL^d^ score, median (IQR)	21 (18-27)	19 (16-22)	27 (22-39)	<.001	

^a^mRS: modified Rankin Scale.

^b^NIHSS: National Institutes of Health Stroke Scale.

^c^PLAN: preadmission comorbidities, level of consciousness, age, and neurological deficit.

^d^ASTRAL: Acute Stroke Registry and Analysis of Lausanne.

The training and test sets consisted of 2885 and 962 patients, respectively. The training set was used to build the document-term matrix and to train the machine learning models. Table S1 in Multimedia Appendix 2 lists the number of unique features and final selected features for each model. The AUCs of full models that used an extra tree classifier for feature selection were higher than the AUCs of those that used penalized logistic regression for feature selection, although the differences did not reach statistical significance. By contrast, penalized logistic regression resulted in higher AUCs than those resulting from extra tree classifiers for simple models, and a significant difference (*P*=.02) was observed for simple model 3. Therefore, machine learning models that used penalized logistic regression for feature selection were used in the following analyses.

**Figure 3:**
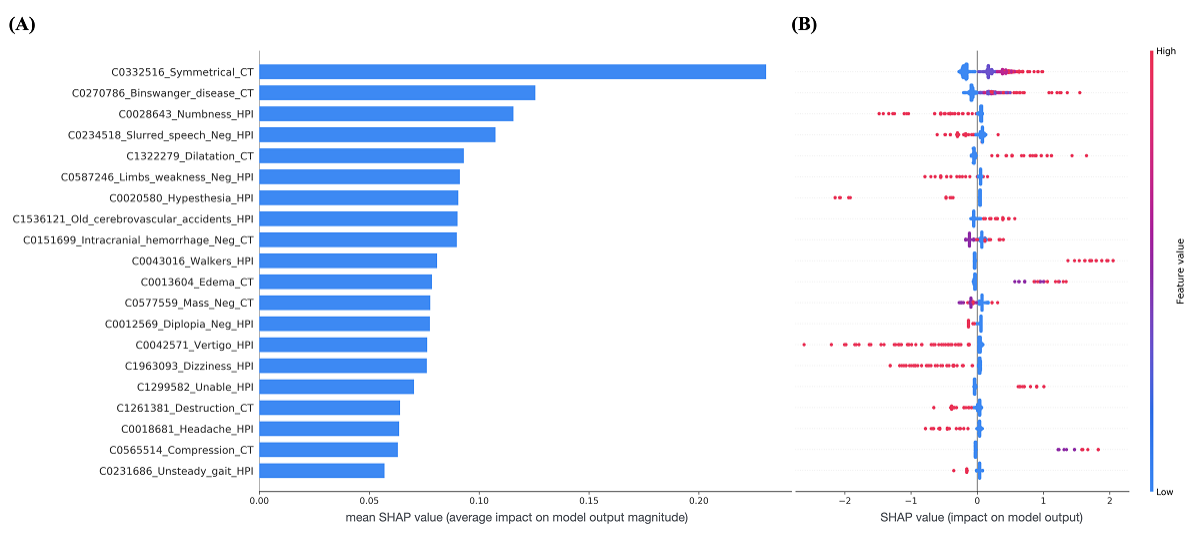
(A) A bar chart showing the top 20 most important features of simple model 2 according to the average absolute SHA*P* values, which indicate the average impact on model output. (B) A bee swarm plot for the top 20 features in which each dot represents an individual patient. A dot’s position on the x-axis indicates the impact that a feature has on the model’s prediction for that patient. The color of the dot specifies the relative value of the corresponding feature (concept). A higher feature value means that the concept appears more times in the clinical text. The prefix before the concept is the concept unique identifier. A negated concept is suffixed with “_Neg”. CT: computed tomography; HPI: history of present illness; SHAP: Shapley additive explanations.

The top 20 features for both good and poor functional outcomes that were used in the simple models are shown in Figure 2. Figure 3 shows the top 20 most important text features from simple model 2; the features are ordered by the average absolute SHA*P* value, which indicates the magnitude of the impact on model output. Figure 3 also presents bee swarm plots showing the magnitude and direction of the effect of each feature according to the SHA*P* value, demonstrating how simple model 2 uses input features to make predictions. For example, when the concepts of *symmetrical*, *Binswanger disease*, or *dilatation* appear in a CT report, the model tends to predict a poor outcome, whereas the model tends to predict a good outcome when an HPI contains the concepts of *numbness* or the negated form of *slurred speech*. Figures S1-S6 in Multimedia Appendix 2 show the bee swarm plots for all text-based models.

**Figure 4:**
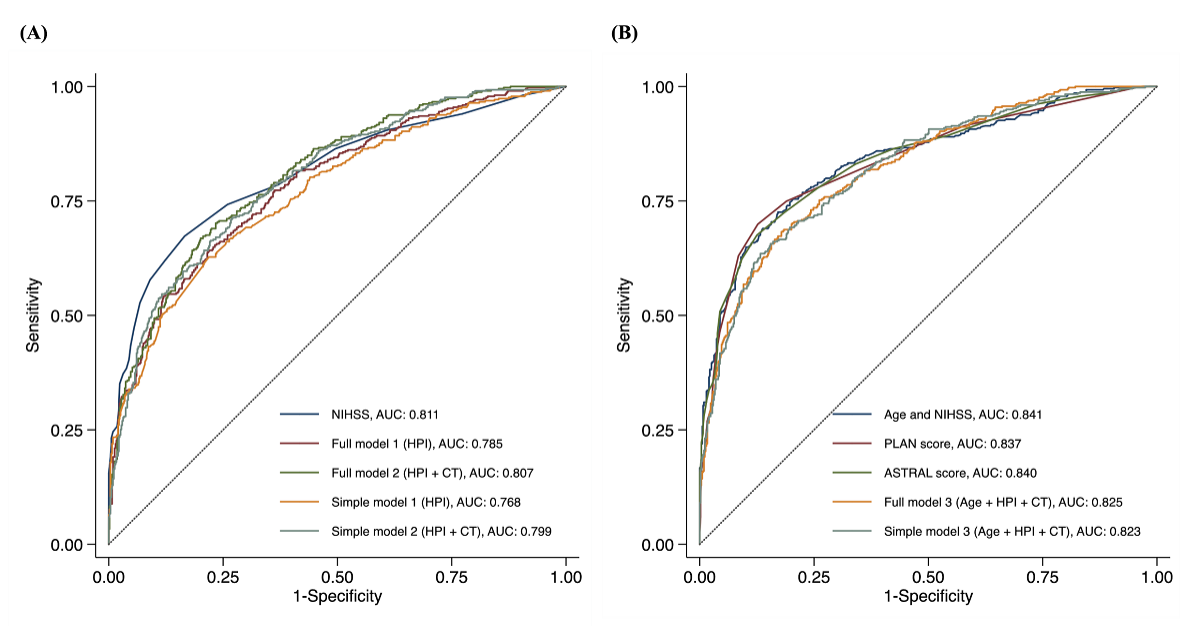
Receiver operating characteristic curves for predicting a poor functional outcome for (A) models without age and (B) models with age. ASTRAL: Acute Stroke Registry and Analysis of Lausanne; AUC: area under the receiver operating characteristic curve; CT: computed tomography; HPI: history of present illness; NIHSS: National Institutes of Health Stroke Scale; PLAN: preadmission comorbidities, level of consciousness, age, and neurological deficit.

Figure 4 illustrates the receiver operating characteristic curves for the six text-based models and the four baseline models trained on the test set. The models are grouped according to whether age is included in the model. Tables S2-S4 in Multimedia Appendix 2 list these models’ AUCs (with 95% CIs) and the *P* values for the pairwise comparison of model performance. Models that included age generally had higher AUC values (range 0.823-0.841) than those of the models that did not include age (range 0.768-0.811). Among the models that did not include age, the AUCs of full model 1 (0.785; 95% CI 0.756-0.814), full model 2 (0.807; 95% CI 0.779-0.834), and simple model 2 (0.799; 95% CI 0.771-0.827) were not significantly different from that of the model that included NIHSS scores (0.811; 95% CI 0.783-0.839; *P*=.11, .78, and .47, respectively). Among the models that included age, the AUCs of full model 3 (0.825; 95% CI 0.799-0.851) and simple model 3 (0.823; 95% CI 0.797-0.850) were also not significantly different from those of the model that included age and NIHSS scores (0.841; 95% CI 0.815-0.867; *P*=.22 and .17, respectively), the model that included the PLAN scores (0.837; 95% CI 0.811-0.863; *P*=.37 and .30, respectively), and the model that included the ASTRAL scores (0.840; 95% CI 0.814-0.866; *P*=.27 and .22, respectively). Table 2 lists the predictive performance of models with and without added information from the clinical text. According to the AUCs (model including age, NIHSS scores, and text: *P*=.002; model include PLAN scores and text: *P*<.001; model including ASTRAL scores and text: *P*=.004), net reclassification improvement indices (all models including text: *P*<.001), and integrated discrimination improvement indices (all models including text: *P*<.001), a statistically significant improvement in predictive performance was achieved when adding information from the clinical text into the baseline models.

**Table 2:** Comparison of the performance of baseline models with or without added information from clinical text.

Model	AUC^a^ (95% CI)	*P* value	NRI^b^, % (95% CI)	*P* value	IDI^c^, % (95% CI)	*P* value
Age and NIHSS^d^ score	0.841 (0.815-0.867)	N/A^e^	N/A	N/A	N/A	N/A
Age and NIHSS score plus text	0.861 (0.837-0.885)	.002	0.427 (0.302-0.551)	<.001	0.042 (0.029-0.054)	<.001
PLAN^f^ score	0.837 (0.811-0.863)	N/A	N/A	N/A	N/A	N/A
PLAN score plus text	0.856 (0.835-0.882)	<.001	0.543 (0.420-0.665)	<.001	0.038 (0.026-0.051)	<.001
ASTRAL^g^ score	0.840 (0.814-0.866)	N/A	N/A	N/A	N/A	N/A
ASTRAL score plus text	0.860 (0.837-0.884)	.004	0.443 (0.318-0.567)	<.001	0.044 (0.031-0.057)	<.001

^a^AUC: area under the receiver operating characteristic curve.

^b^NRI: net reclassification improvement.

^c^IDI: integrated discrimination improvement.

^d^NIHSS: National Institutes of Health Stroke Scale.

^e^N/A: not applicable.

^f^PLAN: preadmission comorbidities, level of consciousness, age, and neurological deficit.

^g^ASTRAL indicates Acute Stroke Registry and Analysis of Lausanne.

## Discussion

### Principal Findings

This study demonstrates that machine learning models based on clinical text may provide an alternative way of prognosticating patients after AIS. Most of the models (3/4, 75%) based on textual data alone performed equally as well as the models based on NIHSS scores, whereas models based on text and patient age had a comparable predictive performance to those of the model based on age and NIHSS scores, the model based on the PLAN scores, and the model base on the ASTRAL scores. In addition, the information extracted from clinical text can be used to improve the predictive performance of existing prognostic scores in terms of the prediction of the 90-day functional outcome.

Previous studies have found that machine learning algorithms had comparable discrimination to or even higher discrimination than that of conventional logistic regression models [10-12]. A possible explanation may be that machine learning algorithms can capture potential nonlinear relationships and handle complex interactions between the input variables and the outcome variable [10,34,35]. On the other hand, the performance of prognostic scores is generally limited by different demographic and risk factor distributions across diverse populations and health care settings [36,37]. By contrast, data-driven models can make predictions without prior knowledge of the real system [38]. The use of machine learning methods may enable each individual site to develop its own prediction models for providing patients with individualized medical decisions and treatments. However, their transferability to different health systems is not guaranteed.

Despite the emergence of machine learning technology as a new tool for prognosticating stroke outcomes, textual data have rarely been analyzed or used in previous machine learning prediction models in the field of stroke medicine [39-44]. By using NLP techniques, information extracted from unstructured text, such as clinical notes or radiology reports, has been used to build machine learning models to identify AIS [39-41] or automate AIS subtype classification [43,44]. One of the advantages of using textual data is that narrative notes are generated during routine health care processes, thus avoiding the extra effort required for data collection and coding. Although structured entry and reporting tools are now available for clinical documentation, health care providers generally prefer to write narrative notes because structured documentation systems can be too awkward to use without impeding clinical workflows and can even result in errors [45,46]. Furthermore, the excessive use of structured data entry in clinical documentation tends to result in the loss of the subtleties in information by standardizing away the heterogeneity across patients [46].

Although only the basic bag-of-words model was used for text representation, this study shows an application of text classification in the development of clinical prediction models. However, a major challenge of this approach is the high dimensionality of the feature space. The large number of features generated by the bag-of-words model may cause problems, such as increased computational complexity, degraded classification performance, and overfitting [21,47]. Feature selection is thus a necessary step for text classification. However, the choice of feature selection methods usually depends on the characteristics of the data and requires trade-offs among multiple criteria, particularly in small samples with high dimensionality [47]. According to our experiments, the two feature selection methods indeed performed slightly differently in different situations.

Another merit of using the bag-of-words approach for text vectorization is the high level of interpretability that can be achieved; this approach allows domain experts to examine each predictor (concept) within its specific context. The patterns that a machine learning model discovers and the explanations for what is observed can be more important than the model’s predictive performance, particularly in medical applications. In this regard, we applied Shapley values to measure the impact of each predictor. Taking the concept *symmetrical* as an example, the reason why this concept tends to be associated with a poor functional outcome (Figure 3) may not be obvious at first glance. The reason became clear when the original text in the CT reports was reviewed. Radiologists generally described subcortical arteriosclerotic encephalopathy as “symmetrical hypodensities in bilateral periventricular regions” and mentioned hydrocephalus as a “symmetrical enlargement of the lateral ventricles.” Both conditions cause a range of impairments in brain function. Consequently, the concept *symmetrical* is commonly found in the CT reports of patients with a poor functional outcome.

### Limitations

This study had some limitations to be addressed. First, although data-driven prediction approaches have their own merits, the relationships discovered from our data do not necessarily indicate causation; therefore, prediction accuracy should never be interpreted as causal validity [48]. Second, this is a single-site study, which may limit the generalizability of study results. Third, although MetaMap was used to extract medical concepts, this study basically adopted the bag-of-words approach to represent clinical text. As such, it disregards the order of concepts and does not capture the contextual dependency between concepts. Furthermore, different kinds of speculative expressions, ranging from completely affirmative to completely nonafﬁrmative, were found in the clinical text. Even though negation detection was used, we did not perform factuality detection. Different types of text representations, such as contextual word embeddings, may be explored in future research. Fourth, the terms and phrases used in clinical documentation may differ across health systems and cultures. This renders the transferability of the machine learning models questionable and may entail that each individual health system has to build its own version of the prediction models and follow a similar process of model development.

### Conclusions

This study demonstrates that by using NLP and machine learning techniques, unstructured clinical text has the potential to improve the early prediction of functional outcomes after AIS. Despite these findings, this does not mean that the machine learning models developed in this study can be directly deployed at other stroke centers. We further suggest that each individual health system develops its own model by applying the proposed methods to its EHRs.

## Appendix

Multimedia Appendix 1 List of common clinical abbreviations and acronyms.

Multimedia Appendix 2 Supplemental material.

## Abbreviations

AIS: acute ischemic stroke
ASCII: American Standard Code for Information Interchange
ASTRAL: Acute Stroke Registry and Analysis of Lausanne
AUC: area under the receiver operating characteristic curve
CT: computed tomography
EHR: electronic health record
HPI: history of present illness
mRS: modified Rankin Scale
NIHSS: National Institutes of Health Stroke Scale
NLP: natural language processing
PLAN: preadmission comorbidities, level of consciousness, age, and neurological deficit
SHAP: Shapley additive explanation
XGBoost: extreme gradient boosting
