# Supplementary material for: Early Prediction of Functional Outcomes After Acute Ischemic Stroke Using Unstructured Clinical Text: Retrospective Cohort Study
Source: JMIR Med Inform. 2022 Feb 17;10(2):e29806. doi: 10.2196/29806 (PMC8895286; doi:10.2196/29806)
Supplement: Multimedia Appendix 2 [file medinform_v10i2e29806_app2.pdf]

Multimedia Appendix 2: Supplemental results

Table S1. Number of features for each machine learning model using different feature selection methods.

		Penalized logistic regression		Extra tree classifier		
Models	Unique features	Selected features	AUC[a] (95% CI[b])	Selected features	AUC (95% CI)	*P*
Full model 1	3892	674	0.785 (0.756–0.814)	1069	0.788 (0.760–0.817)	.53
Full model 2	5341	788	0.807 (0.779–0.834)	1400	0.808 (0.781–0.835)	.71
Full model 3	5342	797	0.825 (0.799–0.851)	1406	0.825 (0.780–0.851)	.91
Simple model 1	40	40	0.768 (0.738–0.799)	33	0.762 (0.732–0.792)	.12
Simple model 2	80	74	0.799 (0.771–0.827)	62	0.794 (0.766–0.822)	.37
Simple model 3	81	78	0.823 (0.797–0.850)	64	0.813 (0.786–0.840)	.02

[a]AUC: area under the receiver operating characteristic curve.
[b]CI: confidence interval.

Table S2. Model performance in terms of AUC.

Models	AUC[a] (95% CI[b])
Models without age	
NIHSS[c]	0.811 (0.783–0.839)
Full model 1	0.785 (0.756–0.814)
Full model 2	0.807 (0.779–0.834)
Simple model 1	0.768 (0.738–0.799)
Simple model 2	0.799 (0.771–0.827)
Models with age	
Age and NIHSS	0.841 (0.815–0.867)
PLAN[d] score	0.837 (0.811–0.863)
ASTRAL[e] score	0.840 (0.814–0.866)
Full model 3	0.825 (0.799–0.851)
Simple model 3	0.823 (0.797–0.850)

[a]AUC: area under the receiver operating characteristic curve.
[b]CI: confidence interval.
[c]NIHSS: National Institutes of Health Stroke Scale.
[d]PLAN: preadmission comorbidities, level of consciousness, age, and neurological deficit.
[e]ASTRAL indicates Acute Stroke Registry and Analysis of Lausanne.

Table S3. *P* values for the pairwise comparison of AUCs[a] between models without age.

Models	NIHSS[b]	Full model 1	Full model 2	Simple model 1	Simple model 2
NIHSS	-	.11	.78	.01	.47
Full model 1		-	.04	.06	.24
Full model 2			-	.001	.34
Simple model 1				-	.003
Simple model 2					-

[a]AUC: area under the receiver operating characteristic curve
[b]NIHSS, National Institutes of Health Stroke Scale.

Table S4. *P* values for the pairwise comparison of AUCs[a] between models with age.

Models	Age and NIHSS[b]	PLAN[c] score	ASTRAL[d] score	Full model 3	Simple model 3
Age and NIHSS	-	.67	.81	.22	.17
PLAN score		-	.75	.37	.30
ASTRAL score			-	.27	.22
Full model 3				-	.84
Simple model 3					-

[a]AUC: area under the receiver operating characteristic curve
[b]NIHSS, National Institutes of Health Stroke Scale.
[c]PLAN: preadmission comorbidities, level of consciousness, age, and neurological deficit.
[d]ASTRAL indicates Acute Stroke Registry and Analysis of Lausanne.

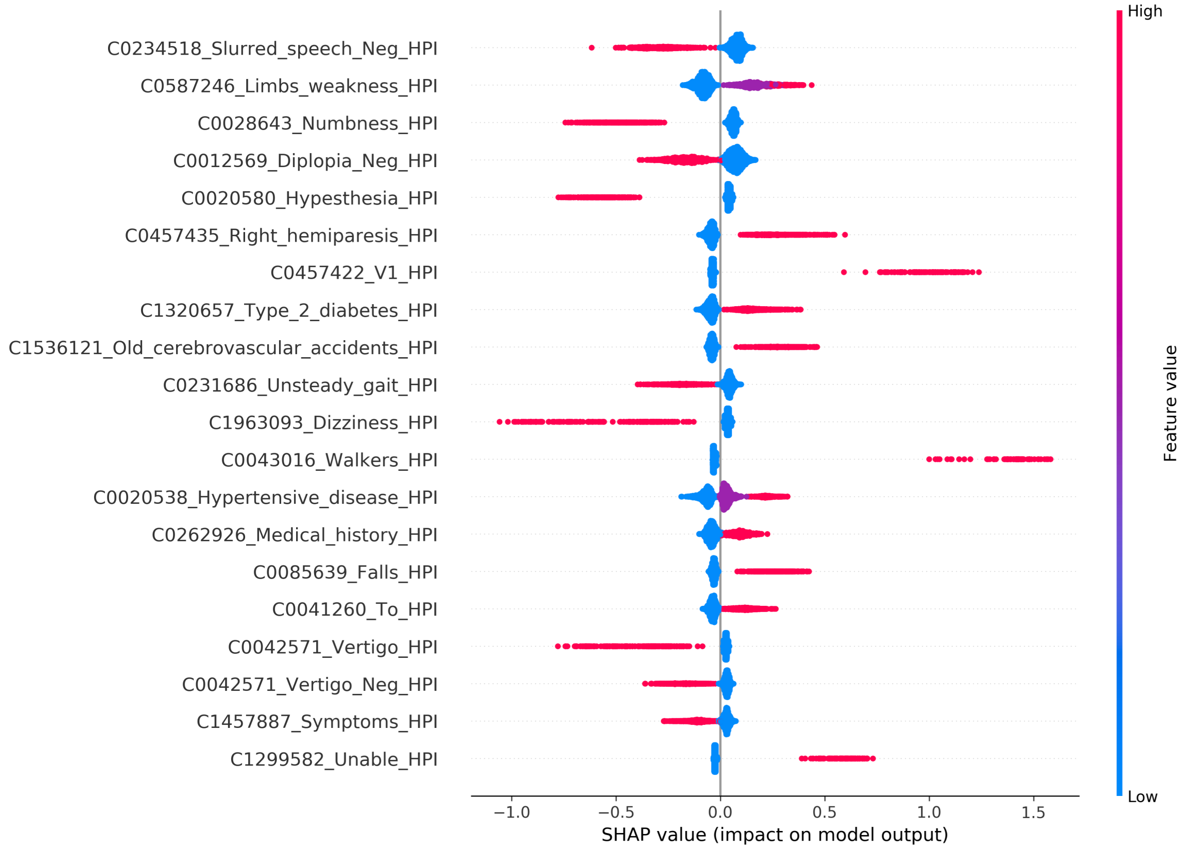

Figure S1. Beeswarm plot for the top 20 features from full model 1. The prefix before the concept is the concept unique identifier. A negated concept is suffixed with "_Neg". HPI: history of present illness.

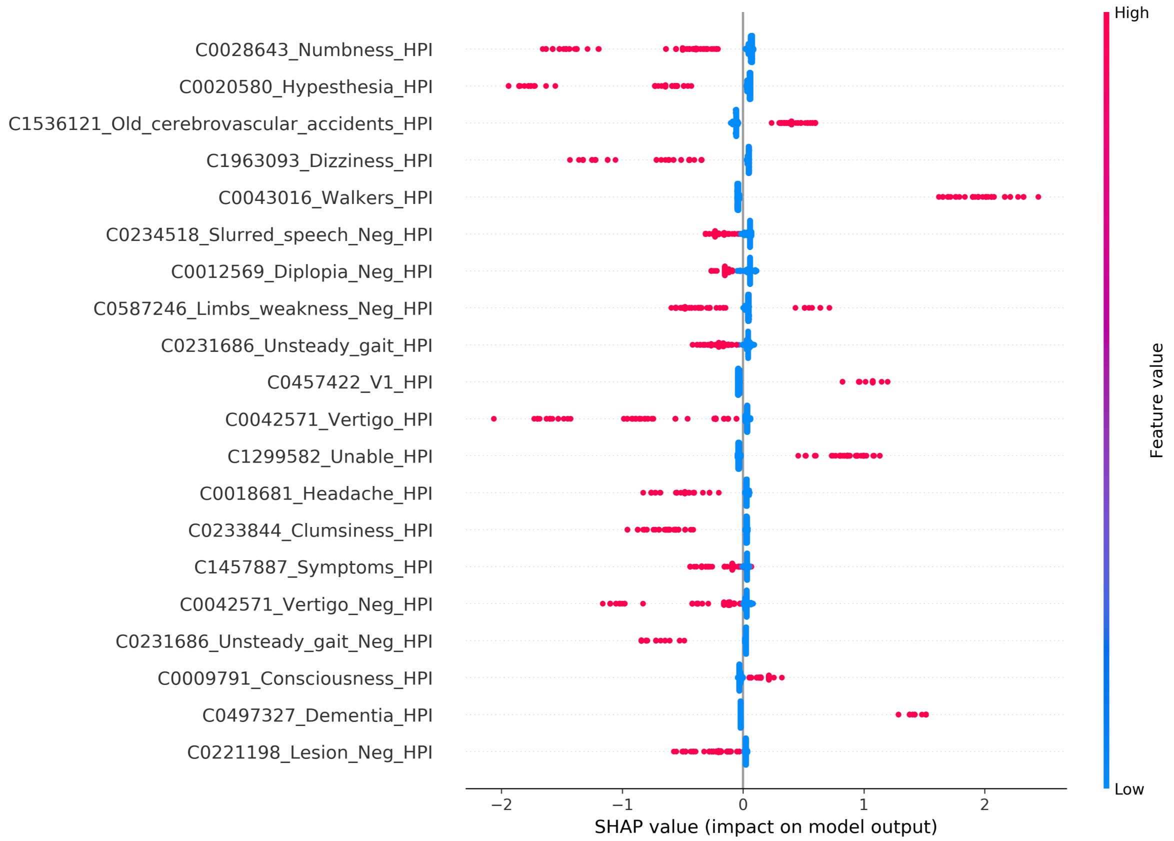

Figure S2. Beeswarm plot for the top 20 features from simple model 1. The prefix before the concept is the concept unique identifier. A negated concept is suffixed with "_Neg". HPI: history of present illness.

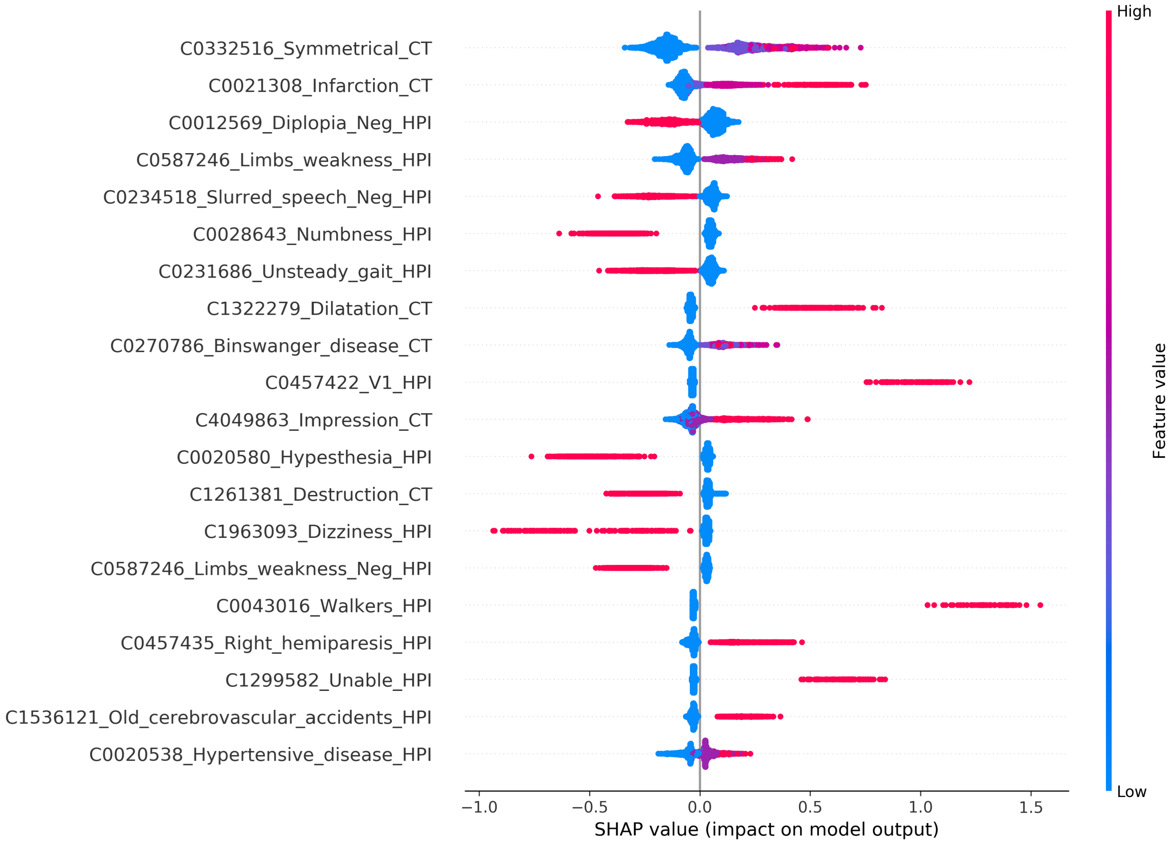

Figure S3. Beeswarm plot for the top 20 features from full model 2. The prefix before the concept is the concept unique identifier. A negated concept is suffixed with "_Neg". CT: computed tomography. HPI: history of present illness.

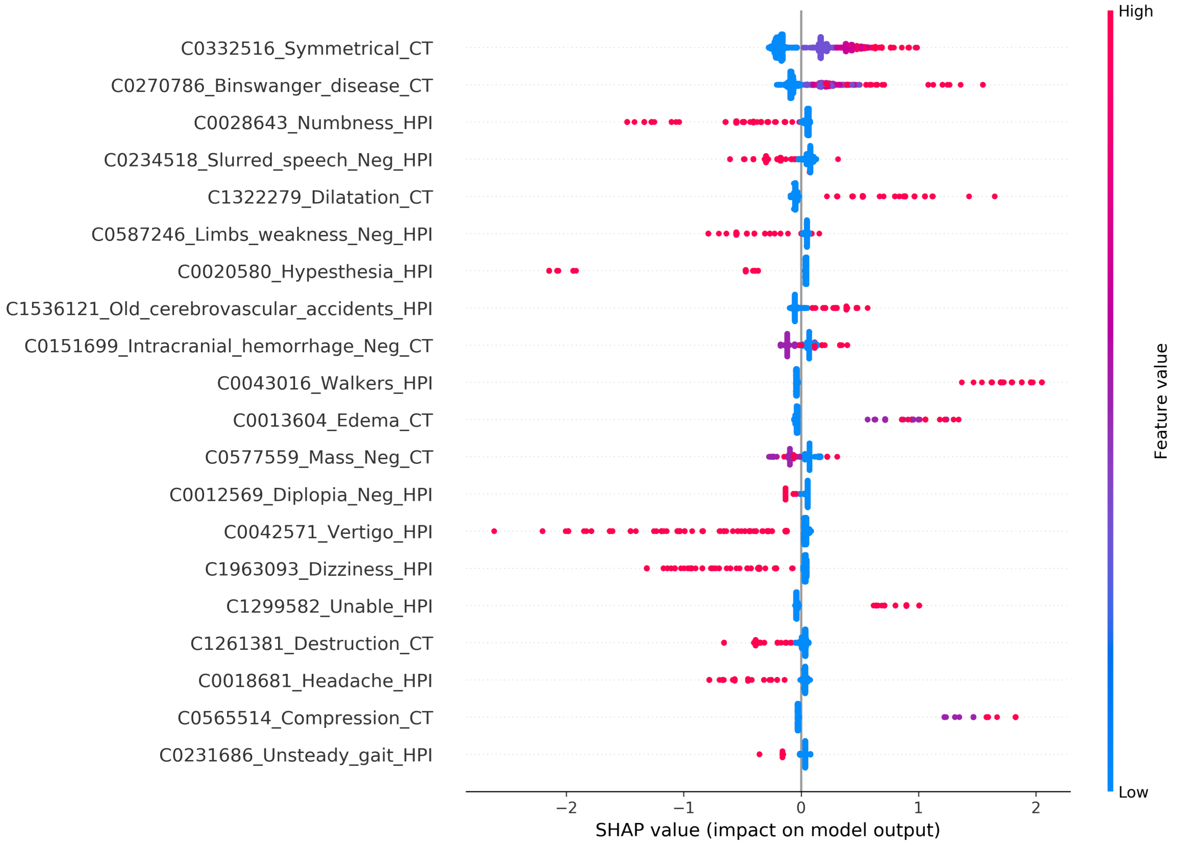

Figure S4. Beeswarm plot for the top 20 features from simple model 2. The prefix before the concept is the concept unique identifier. A negated concept is suffixed with "_Neg". CT: computed tomography. HPI: history of present illness.

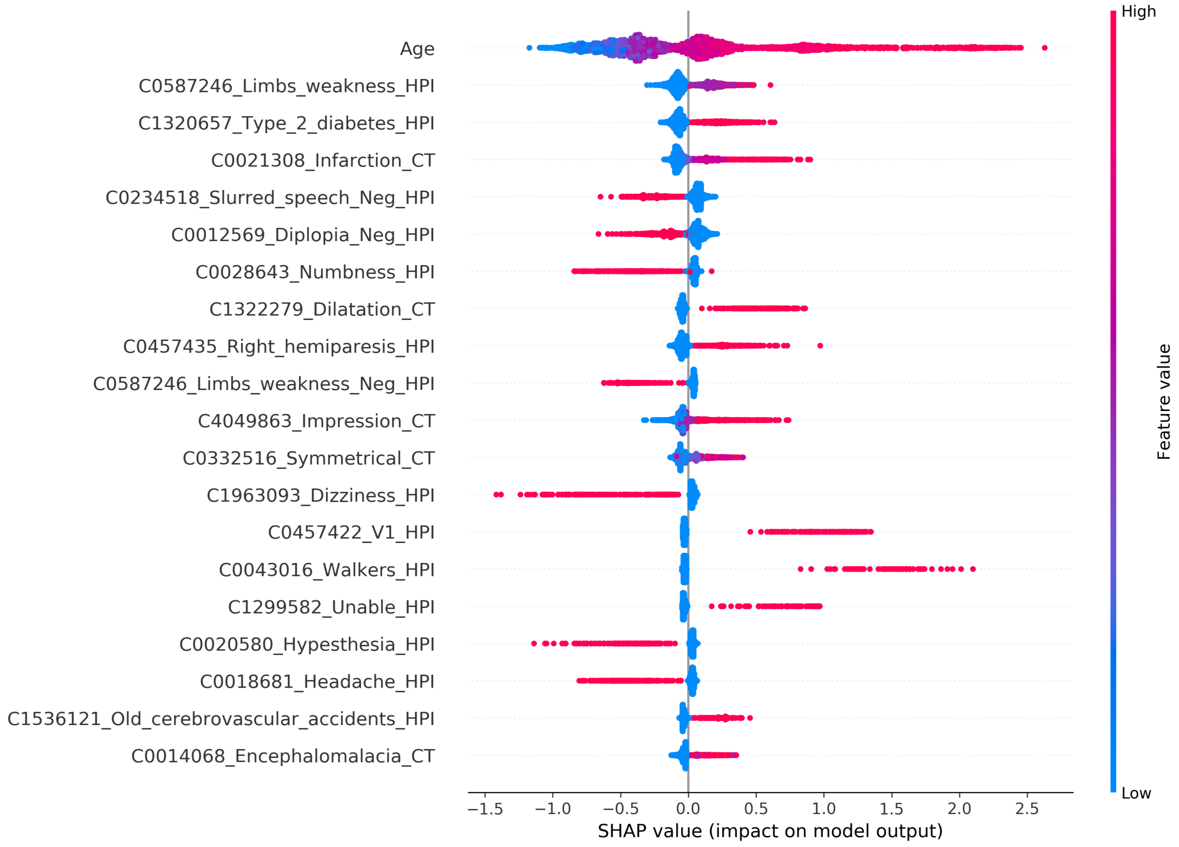

Figure S5. Beeswarm plot for the top 20 features from full model 3. The prefix before the concept is the concept unique identifier. A negated concept is suffixed with “_Neg”. CT: computed tomography. HPI: history of present illness.

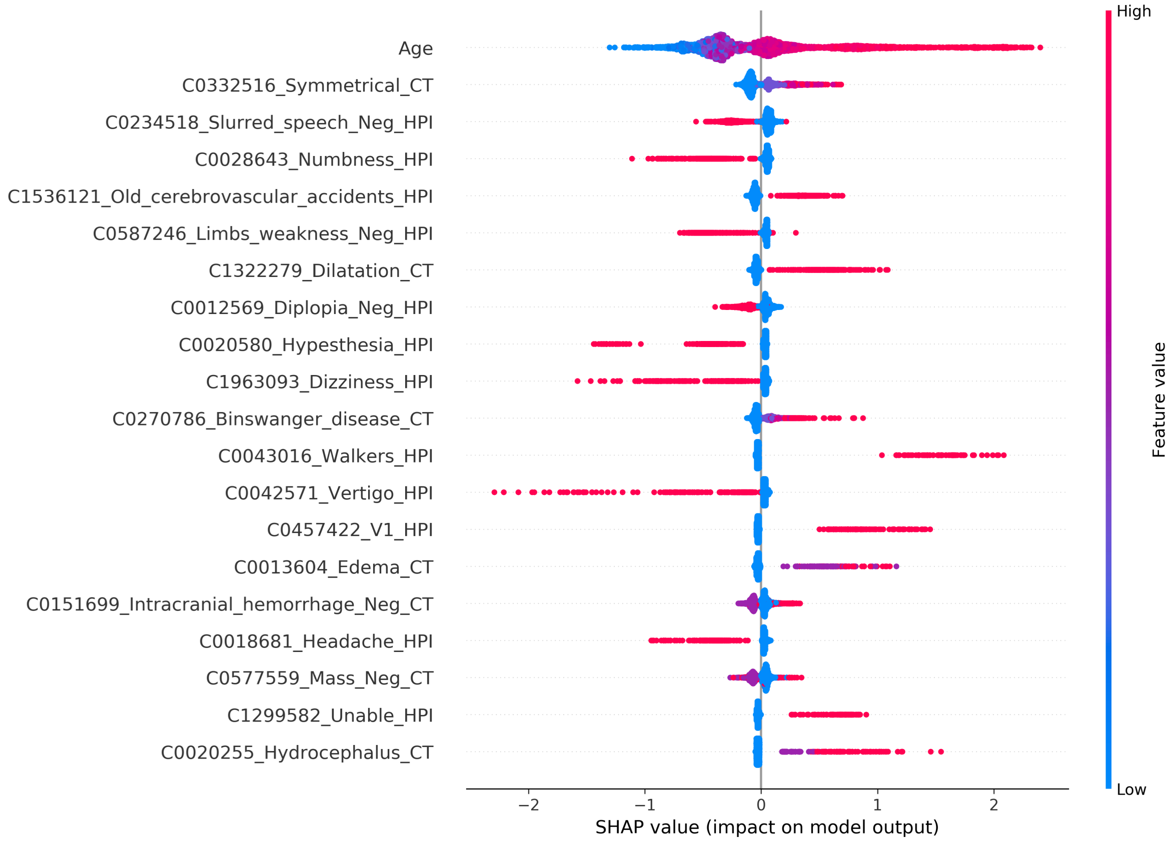

Figure S6. Beeswarm plot for the top 20 features from simple model 3. The prefix before the concept is the concept unique identifier. A negated concept is suffixed with “_Neg”. CT: computed tomography. HPI: history of present illness.
